# Altered diffusion tensor imaging measurements in aged transgenic Huntington disease rats

**DOI:** 10.1007/s00429-012-0427-0

**Published:** 2012-05-23

**Authors:** Bjørnar T. Antonsen, Yi Jiang, Jelle Veraart, Hong Qu, Huu Phuc Nguyen, Jan Sijbers, Stephan von Hörsten, G. Allan Johnson, Trygve B. Leergaard

**Affiliations:** 1 Center for Molecular Biology and Neuroscience, Institute of Basic Medical Sciences, University of Oslo, Oslo, Norway; 2 Center for In Vivo Microscopy, Department of Radiology, Duke University Medical Center, Durham, NC USA; 3 Vision Lab, Department of Physics, University of Antwerp, Antwerp, Belgium; 4 Department of Medical Genetics, University of Tübingen, Tübingen, Germany; 5 Department for Experimental Therapy, Franz-Penzoldt-Center, Friedrich-Alexander-University Erlangen-Nürnberg, Erlangen-Nürnberg, Germany

**Keywords:** Basal ganglia, Diffusion magnetic resonance imaging, Hippocampus, Magnetic resonance imaging, Neurodegenerative disease, Neuroimaging, Transgenic rat model

## Abstract

Rodent models of Huntington disease (HD) are valuable tools for investigating HD pathophysiology and evaluating new therapeutic approaches. Non-invasive characterization of HD-related phenotype changes is important for monitoring progression of pathological processes and possible effects of interventions. The first transgenic rat model for HD exhibits progressive late-onset affective, cognitive, and motor impairments, as well as neuropathological features reflecting observations from HD patients. In this report, we contribute to the anatomical phenotyping of this model by comparing high-resolution ex vivo DTI measurements obtained in aged transgenic HD rats and wild-type controls. By region of interest analysis supplemented by voxel-based statistics, we find little evidence of atrophy in basal ganglia regions, but demonstrate altered DTI measurements in the dorsal and ventral striatum, globus pallidus, entopeduncular nucleus, substantia nigra, and hippocampus. These changes are largely compatible with DTI findings in preclinical and clinical HD patients. We confirm earlier reports that HD rats express a moderate neuropathological phenotype, and provide evidence of altered DTI measures in specific HD-related brain regions, in the absence of pronounced morphometric changes.

## Introduction

Huntington disease (HD) is a late manifesting inheritable neurodegenerative disease caused by an expanded trinucleotide repeat in the coding region of the HD gene (HDCRG 1993; Vonsattel and DiFiglia 1998). The clinical manifestation of disease includes motor, emotional, cognitive impairments and premature death (Harper 1996). The neuropathological hallmark of the disease is atrophy and neuronal loss in the striatum, but neurodegeneration is also seen in other parts of the basal ganglia, as well as in the cerebral cortex, thalamus, subthalamic nucleus, hypothalamus, hippocampus, and cerebellum (Tellez-Nagel et al. 1974; Jeste et al. 1984; Vonsattel et al. 1985; Cudkowicz and Kowall 1990; Hedreen et al. 1991; Spargo et al. 1993; Sapp et al. 1997; Vonsattel and DiFiglia 1998; MacDonald and Halliday 2002; Gardian and Vecsei 2004). Rodent models of HD provide useful tools to investigate the pathophysiology of HD and evaluate potential new therapeutic approaches (Vonsattel 2008; Simpson et al. 2010; Urbach et al. 2010). The first transgenic rat model of HD carries a truncated huntingtin cDNA fragment with 51 CAG repeats under control of the native rat huntingtin promoter (von Hörsten et al. 2003). When compared with genetic mouse models of HD, the relative larger size of the rat brain is advantageous for application of translational imaging tools, such as positron emission tomography (PET) and magnetic resonance imaging (MRI; von Hörsten et al. 2003; Blockx et al. 2011, 2012).

HD rats exhibit progressive emotional disturbance, cognitive decline, and motor deficits (von Hörsten et al. 2003; Cao et al. 2006; Nguyen et al. 2006; Brooks et al. 2009; Faure et al. 2010; Hohn et al. 2011) as well as loss of coordinated striatal neuronal firing patterns (Miller et al. 2010) and altered prefrontostriatal functional plasticity (Hohn et al. 2011). Histopathological investigations have revealed striatal atrophy (Kantor et al. 2006; Nguyen et al. 2006), neuronal loss in the basal forebrain (Bode et al. 2008), as well as nuclear aggregates of mutant huntingtin and degenerative changes in the ventral striatopallidal system, extended amygdala and subventricular zone (Nguyen et al. 2006; Petrasch-Parwez et al. 2007). PET and structural MRI investigations of HD rats have shown altered glucose metabolism and age-related changes in the striatum (von Hörsten et al. 2003), longitudinal PET and diffusion MRI investigations have shown differential aging effects, but otherwise modest expression of disease in 12 months old HD rats (Blockx et al. 2011), and more recently an in vivo diffusion kurtosis imaging study measured altered diffusion parameters in the cerebral cortex and dorsal striatum, indicating abnormal neurodevelopment in young HD rats (Blockx et al. 2012).

Diffusion tensor imaging (DTI) has emerged as a sensitive tool for detection of altered tissue integrity at both preclinical and clinical stages of HD (Mascalchi et al. 2004; Reading et al. 2005; Rosas et al. 2006; Seppi et al. 2006; Bohanna et al. 2008; Kloppel et al. 2008; Douaud et al. 2009; Mandelli et al. 2010; Della Nave et al. 2010; Rosas et al. 2010; Sritharan et al. 2010), and has been confirmed as a promising diagnostic tool for monitoring neuropathological changes in rat models of HD (Blockx et al. 2011; Van Camp et al. 2012). The in vivo imaging study of transgenic HD rats by Blockx et al. (2011) was, however, limited by voxel resolutions insufficient to discriminate individual basal ganglia regions, and the apparent subtle neuropathology observed in 12 months old animals.

We here contribute to the further anatomical phenotyping of this model by comparing high-resolution ex vivo diffusion tensor imaging (DTI) measurements obtained in 18 months old transgenic HD rats and wild-type controls. By region of interest analyses supplemented with voxel-based statistics, we demonstrate significantly altered DTI measurements in specific brain regions that are associated with clinical manifestations of HD. We confirm that DTI measures are potentially useful correlates of HD-related changes in these transgenic rats.

## Materials and methods

### Animals

Five male transgenic HD rats carrying a truncated huntingtin cDNA fragment with 51 CAG repeats under control of the native rat huntingtin promoter (von Hörsten et al. 2003), and five age and sex matching wild-type control animals were used. The animals derive from a Sprague–Dawley (SD) founder oocyte (Max Delbrück Center, Berlin-Buch, Germany), by classical pronucleus microinjection using a transgene with the coding sequence of a truncated (t), mutant (m), huntingtin (HTT) protein bearing 51CAG repeats (human PCR product). The animals were subsequently inbred by strict brother × sister mating for 26 generations by Dr. Stephan von Hörsten (SvH). The SD/MdcSvh-Tg (tmHTT51CAG) animals, in the following referred to as transgenic HD rats, were bred and genotyped at the Franz-Penzoldt-Center, Experimental Therapy, Friedrich-Alexander-University of Erlangen-Nürnberg, Germany. After genotyping, the rats were housed in genotype-matched pairs in accordance with FELASA recommendations, and kept under a 12:12 h light:dark cycle with food (Altromin lab chow pellets, Altromin standard diet: 1320; Lage, Germany) and water ad libitum. All animal procedures were approved by the local institutional animal welfare committees at the Universities of Erlangen-Nürnberg and Oslo, and were in compliance with National Institutes of Health and European Community guidelines for the use and care of laboratory animals.

### Sample preparation

The rats were killed at ~18 months of age, well beyond the age when HD-like symptoms are expected to occur, as demonstrated in several earlier behavioral investigations of transgenic HD rats (von Hörsten et al. 2003; Cao et al. 2006; Kantor et al. 2006; Nguyen et al. 2006; Bode et al. 2008; Brooks et al. 2009). Specimens were prepared for active staining according to Johnson et al. (2002) with a fixative mixed with a contrast agent to enhance the MRI signal. Following a brief inhalation induction with 4 % isoflurane (Abbott Laboratories, Illinois, USA), animals were deeply anesthetized by intraperitoneal injection of sodium pentobarbital (50 mg/kg) and transcardially perfused with 120 ml of a mixture of 0.9 % saline, ProHance R (10:1 v:v; gadoteridol, Bracco Diagnostics, Inc, Princeton, NJ), and heparin (5,000 IE units/ml; Leo Pharma A/S, Ballerup, Denmark), followed by 120 ml of freshly prepared 4 % paraformaldehyde with ProHance (10:1 v:v). Animal heads were isolated, stored in 0.9 % saline with ProHance (10:1 v:v), and shipped in pressure and temperature resistant containers to the Duke Center for In Vivo Microscopy for MRI scanning. During all procedures brains were left in situ within the cranium to limit physical distortions.

### MRI acquisition

Diffusion weighted images were acquired between 10 and 24 days after animal sacrifice at the Duke Center for In Vivo Microscopy using a Magnex 7.0 T/210 mm bore magnet controlled by GE EXCITE consoles. Specimens were imaged in a solenoid RF coil fabricated from a continuous sheet of high-frequency microwave substrate (Roger Corp, Rogers, Ct.). A diffusion-weighted spin-echo pulse sequence with extended dynamic range (Johnson et al. 2007) was used to acquire 3D volume images (FOV = 45 × 22.5 × 22.5 mm^3^, TR/TE = 100/15.6 ms, NEX = 2). Diffusion encoding was performed using a pair of half-sine gradient pulses (δ = 3.2 ms/Δ = 8.3 ms), using *b* = 800 s/mm^2^. A reduced encoding DTI methodology (Jiang et al. 2004) was employed, such that each dataset consisted of a fully encoded 512 × 256 × 256 (readout × phase × slice) matrix-size *b*0 (i.e., *b* ≈ 0) and 12 reduced encoded (512 × 128 × 128) diffusion-weighted images (DWI) sensitized in each of an optimized set of 12 directions (Papadakis et al. 1999). Each reduced encoded diffusion-weighted image was reconstructed to 512 × 256 × 256 matrix size by a corrected keyhole algorithm (Jiang and Hsu 2005) with the *b*0 image as the constraining reference, resulting in 88 μm isotropic resolution for each image. The acquisition time for one complete DTI dataset was approximately 18 h. A circulating water cooling system was used in the gradient coils, and a temperature increase of up to 3 °C was observed during the acquisition in all cases. This was not corrected during post-processing. An RF refocused spin-echo image with the same FOV and resolution was acquired with TR = 50 ms, TE = 5 ms, NEX = 1. Since active staining with Prohance reduces the T1 of all the tissues to <100 ms, this sequence produces anatomical images similar to those one would obtain with proton density weighting in unstained tissues (Johnson et al. 2007).

### Image processing

To remove misregistration due to residual eddy-current induced image distortion, all DWI, *T*_1_-weighted, and *T*_2_*-weighted images were co-registered to the corresponding *b*0 image using a mutual information based shift-only registration program (Mistry and Hsu 2006), which was performed in 64-bit MATLAB (The MathWorks, Natick, MA) with Fourier transform-based deformations to minimize blurring effect arising from interpolation. At each voxel, diffusion tensor was calculated with multivariate linear fitting in 64-bit DTIStudio (H. Jiang and S. Mori, Johns Hopkins University, Kennedy Krieger Institute; Jiang et al. 2006). Derived diffusion parameters, such as fractional anisotropy (FA), mean diffusivity (MD), axial diffusivity (AD), and radial diffusivity (RD), were computed from the three eigenvalues after tensor decomposition (Le Bihan et al. 2001).

### Region of interest analysis

**Fig. 1:**
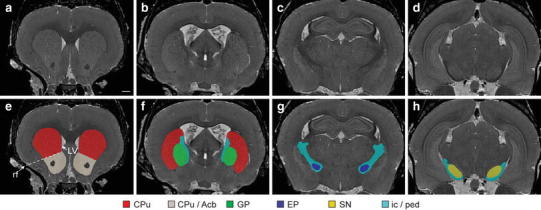
Anatomical segmentation of structural MRI images. Selected coronal slices from high-resolution *T*_1_ weighted images used for anatomical delineation of regions of interest. The dorsal and ventral striatum were arbitrarily subdivided using a line connecting the ventral tip of the lateral ventricle (LV) and the rhinal fissure (rf). *CPu* caudate−putamen complex (dorsal striatum), *CPu/Acb* caudate–putamen complex and accumbens nucleus (ventral striatum), *EP* entopeduncular nucleus, *GP* globus pallidus, *ic* internal capsule, *ped* cerebral peduncle, *SN* substantia nigra. *Scale bar* 1 mm

We first conducted a hypothesis-driven analysis, in which measurements were obtained from a set of anatomically defined grey and white matter regions thought to be affected in HD, as well as a few control regions. Bilateral regions of interest (ROIs) were manually delineated (Fig. 1) on the basis of *T*_1_ weighted and DTI contrast (FA and principal eigenvector orientation) using the ITK-SNAP (version 1.6; http://www.itksnap.org; Yushkevich et al. 2006) and Amira (version 5.4.4., Visage Imaging Inc, San Diego, CA) software packages. The ROIs included several grey (dorsal and ventral striatum; globus pallidus, GP; entopeduncular nucleus EP; substantia nigra, SN) and white matter structure (the internal capsule, ic; the anterior part of the corpus callosum, cc) as well as the anterior portion of the lateral ventricles (LV). The employed anatomical terminology is derived from the sixth edition of the rat brain atlas of Paxinos and Watson (2007). Two standard rat brain atlases were used as a reference (Swanson 2004; Paxinos and Watson 2007), and additional predefined anatomical criteria were employed to close anatomical boundaries when these were not unequivocally visible in *T*_1_ or DTI images (see Veraart et al. 2011a for further details). This concerned the posterior limits of the striatum and globus pallidus which were arbitrarily set at the most anterior level containing the distinct CA3 field of the hippocampus in coronal slices. The segmentations of the lateral ventricles were truncated at the same level. Furthermore, the ventral and dorsal striatum were arbitrarily subdivided by drawing a line between the rhinal fissure and the ventral tip of the lateral ventricle (Fig. 1a; Ingham et al. 1998; Van de Berg et al. 2000). Thus, in our analysis, the dorsal striatum includes the majority of the caudate–putamen complex, while the ventral striatum includes the ventral part of the caudate–putamen complex and the core and shell of the accumbens nucleus. As additional control, average DTI measurements were also sampled bilaterally from square ROIs positioned well within the anatomical boundaries of the pontine nuclei (PN) and inferior colliculus (IC) as well as the longitudinal fibers of the pontine nuclei (lfp). These regions are not known to be associated with the HD pathophysiological process (Vonsattel et al. 1985; Sapp et al. 1997; Vonsattel and DiFiglia 1998), although neuropathological markers for HD have been reported in tectum of old transgenic HD rats (Nguyen et al. 2006). Average DTI metrics (FA, MD) were extracted using Matlab, and regional volumes calculated from the number of voxels included in the different regions. Descriptive statistics and statistical comparisons by two-tailed *t* test for equality of means were computed using the PASW Statistics 18 software (SPSS Inc). A *p* < 0.05 was considered statistically significant.

### Voxel-based statistics

We also conducted a supplementary voxel-based statistical comparison (VBS). To this end, DTI maps were initially normalized to an arbitrarily chosen reference dataset, i.e. one wild-type control animal, by an affine registration. The affine transformation matrix was estimated by maximizing the mutual information between the FA maps of the reference and floating data sets (Maes et al. 1997). To increase the VBS reliability, residual local image misalignments were corrected by warping all subjects onto a study-specific population-based DTI atlas using a non-rigid co-registration technique, which was optimized for the alignment of multiple DTI information components (Van Hecke et al. 2007, 2008). The images were modeled as a viscous fluid whose deformation was driven by a simplified Navier–Stokes equation (D’Agostino et al. 2003). The co-registration algorithm took full advantage of the relevant information that was encoded in DT images, particularly the tensor orientation, thus enabling a better alignment of different WM structures. During the construction of the study-specific population-based DTI atlas (Veraart et al. 2011a) the magnitude of the deformation fields needed to warp the different images to the atlas and the bias to a specific topology were minimized. FA and MD maps were smoothed with an anisotropic Gaussian kernel of 0.6 mm full width half maximum. Comparisons of FA and MD maps were computed in SPM using a parametric two-sample *t* test at each voxel, respectively. For each of the DTI parameters significance maps showing clusters of >25 voxels that are significantly different between the groups at the level of *p* < 0.01 (cfr. Van Camp et al. 2009; Qi et al. 2010), were visualized using Amira.

## Results

High-resolution, contrast enhanced ex vivo structural MRI and DTI measurements were successfully obtained from 9 rats (5 transgenic HD, and 4 WT controls), while 1 WT acquisition failed. The animals were investigated at an age of ~18 months, well beyond the age when significant behavioral and morphological changes are demonstrated in this model (Nguyen et al. 2006; Cao et al. 2006; Kantor et al. 2006; Temel et al. 2006; Brooks et al. 2009; Faure et al. 2010; Miller et al. 2010; Hohn et al. 2011).

### Morphometric analysis

**Fig. 2:**
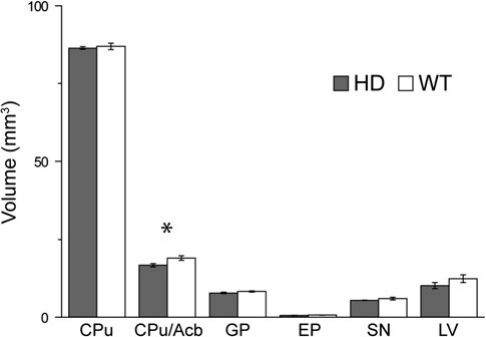
Volumetric analysis of the basal ganglia and lateral ventricle. *Column graphs* showing average volumes of the segmented regions of interest. A reduced average volume was seen in the ventral striatum of transgenic HD rats (*p < 0.05). *CPu* caudate–putamen complex (dorsal striatum), *CPu/Acb* caudate–putamen complex and accumbens nucleus (ventral striatum), *EP* entopeduncular nucleus, *GP* globus pallidus, *LV* lateral ventricle, *SN* substantia nigra, *HD* transgenic HD rats, *WT* wild-type rats

To first evaluate for presence of atrophic changes in transgenic HD rats, we compared the volumes of the segmented basal ganglia regions (dorsal striatum; ventral striatum; globus pallidus, GP; entopeduncular nucleus, EP; substantia nigra, SN; Fig. 1) and the lateral ventricles between the tgHD and WT groups. A significant volume reduction of ~12 % was found in the ventral striatum of transgenic HD rats (*p* = 0.036), while no significant differences were seen in the regions investigated (Fig. 2). We further observed an insignificant trend towards reduced lateral ventricle volumes in HD rats. We conclude that there is evidence for moderate atrophy of the ventral striatum in these transgenic HD animals, but otherwise not for pronounced basal ganglia atrophy or ventricular enlargement.

### DTI measurements

To determine if altered DTI measurements occurred in brain regions typically associated with HD, we compared the DTI measures fractional anisotropy (FA), mean diffusivity (MD), axial diffusivity (AD), and radial diffusivity (RD), between the transgenic HD and wild-type groups, using (1) a hypothesis-driven approach in which average values were compared for anatomically defined ROIs, and (2) an exploratory approach using voxel-based statistics.

#### Comparison of average DTI measures in regions of interest

**Fig. 3:**
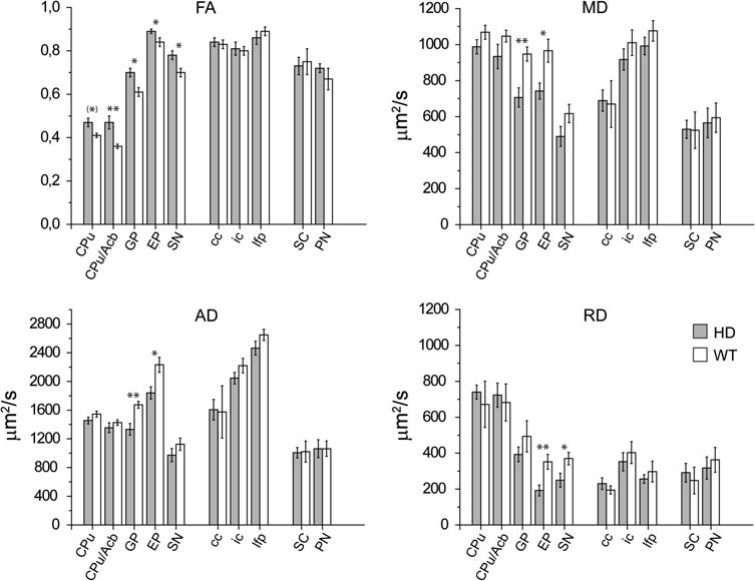
Comparison of DTI parameters in regions of interest. *Column graphs* showing average bilateral fractional anisotropy (FA), mean diffusivity (MD), axial diffusivity (AD), and radial diffusivity (RD) values in a priori segmented regions of interest. A significantly increased average FA value was seen in the ventral striatum, globus pallidus, entopeduncular nucleus, and substantia nigra of transgenic HD rats (*p < 0.05; **p < 0.0125, significance level after Bonferroni’s correction). The increased average FA value in the dorsal striatum was close to significance (^(^*^)^ *p* < 0.1). No FA differences were seen in the other regions investigated. Significantly reduced MD and AD values were found in the globus pallidus (**p < 0.0125) and entopeduncular nucleus (*p < 0.05) of the transgenic HD rats, while significantly reduced RD was observed in the entopeduncular nucleus (**p < 0.0.125). A RD reduction close to significance was also seen in the substantia nigra (^(^*^)^ *p* < 0.1). Similar trends of reduced diffusivity were also observed in the striatum, substantia nigra, and internal capsule, however not reaching significance. No MD, AD, or RD differences were seen in the other regions investigated. *cc* Corpus callosum, *CPu* caudate–putamen complex (dorsal striatum), *CPu/Acb* caudate–putamen complex and accumbens nucleus (ventral striatum), *EP* entopeduncular nucleus, *GP* globus pallidus, *ic* internal capsule, *lfp* longitudinal fibers of the pons, *PN* pontine nuclei, *SC* superior colliculus, *SN* substantia nigra, *HD* transgenic HD rats, *WT* wild-type rats

To test the hypothesis that DTI changes occur in the striatum and its main axonal projection targets (the lateral and medial globus pallidus and the substantia nigra; Parent et al. 2000; Gerfen, 2005), we compared average FA and diffusivity measures (MD, AD, and RD) obtained from these regions, as well as the internal capsule (ic), through which striatofugal fibers pass. We also included the anterior part of the corpus callosum (cc), in which DTI changes have been reported in HD patients (Rosas et al. 2006). For additional control, we further compared FA and diffusivity values sampled from grey matter regions in the superior colliculus (SC), and pontine nuclei (PN), white matter regions in the longitudinal fibers of the pons (lfp), and the lateral ventricles (LV). In the transgenic HD rats, the average FA values were significantly increased in the ventral striatum (*p* = 0.012), GP (*p* = 0.017), EP (*p* = 0.030), and SN (*p* = 0.028), while the average MD values were significantly decreased in the GP and EP (*p* = 0.011 and 0.021, respectively). Similarly, AD was significantly reduced in the GP and EP (*p* = 0.012 and 0.022, respectively), while RD was reduced in the EP and SN (*p* = 0.015 and 0.059, respectively). When applying Bonferroni correction for multiple comparisons of the ROI analysis (*p* < 0.0125) significant changes were still observed in the ventral striatum, GP and EP. It should, however, be noted that these corrections probably are too conservative here, since the measured parameters have complex dependencies. No changes in average diffusivity were found in the dorsal or ventral striatum. In white (cc, ic, and lfp) and grey matter (SC, PN) regions, average FA and diffusivity measurements were similar between the two groups. Figure 3 shows a graphical overview of these comparisons.

#### Voxel-based statistical analysis of DTI measures

Our ROI analyses were supplemented with an exploratory voxel-based statistical survey of the entire brain. For this analysis, DTI images were co-registered using a non-rigid algorithm on basis of the FA maps to correct for morphological differences. At each voxel, parametric two-sample Student’s *t* tests were performed to assess differences in DTI parameters (FA, MD, AD, RD) between the transgenic HD and wild-type animals across the entire brain. Statistical maps showing significantly altered FA and diffusivity values were generated on basis of the co-registered DTI images. Adopting values from two previous investigations employing similar analyses (Van Camp et al. 2009; Qi et al. 2010) we set the threshold for statistical significance at *p* < 0.01, and chose a cluster size threshold of 25 voxels to minimize false-positive observations.

**Fig. 4:**
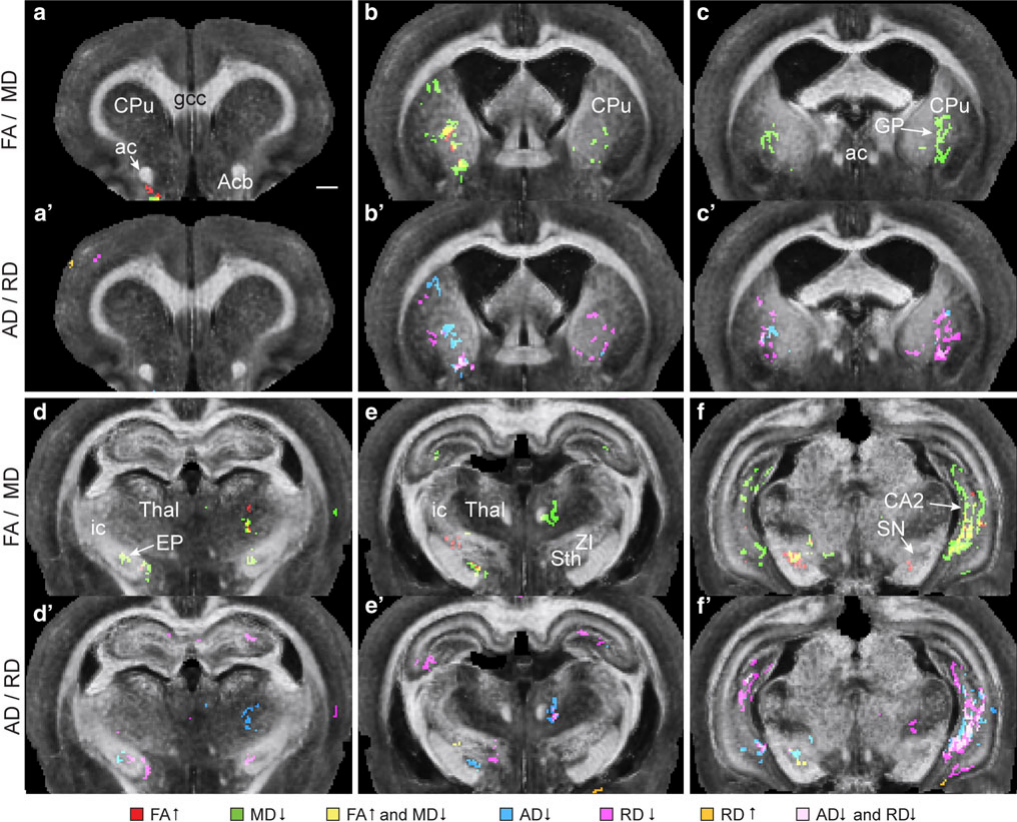
Diffusion tensor significance maps showing voxels with altered fractional anisotropy and mean diffusivity (**a**–**f**), or with altered axial- and radial diffusivity (**a**′–**f**′). The maps are presented in coronal slices (viewed from posterior) and show voxels with significantly different DTI metrics in HD rats, at a statistical significance level of *p* < 0.01, with a cluster threshold of 25 voxels. Significant DTI changes are seen in the ventral striatum (**a**, **a**′), dorsal striatum and globus pallidus (**b**, **b**′, **c**, **c**′), thalamus and medial globus pallidus (**d**, **d**′, **e**, **e**′), subthalamic region (**e**, **e**′), substantia nigra (**f**, **f**′) and the CA2 region of the hippocampus (**f**, **f**′). *ac* anterior commissure, *Acb* accumbens nucleus, *CA2* field cornu ammonis 2 of the hippocampus, *CPu* caudate putamen complex, *EP* entopeduncular nucleus, *gcc* genu of the corpus callosum, *GP* globus pallidus, *SN* substantia nigra, *STh* subthalamic nucleus, *tgHD* transgenic HD rats, *Thal* thalamus, *WT* wild-type rats, *ZI* zona incerta. *Scale bar* 1 mm

The significance maps revealed clusters of increased FA, decreased MD, AD, and RD in the basal ganglia (ventral and dorsal striatum, GP, EP, SN; Fig. 4) and subthalamic region (Fig. 4e, e′), as well as in larger parts of the CA2 region of the hippocampus (Fig. 4f, f′) and a few small regions in the ventrolateral and ventral posterolateral thalamic nucleus (Fig. 4d, d′, e, e′). A few clusters with increased RD values were also seen in the ventral striatum, EP, and SN. In addition, some minor FA/MD changes were observed in a limited part of the ic, close to the EP (Fig. 4c), and a some clusters of decreased and increased RD were found in the frontal and parietal cerebral cortex (Fig. 4a′). No other cortical changes were seen. The voxels with increased FA and altered diffusivity were typically co-located and partly overlapping (Fig. 4). The number of voxels with altered diffusivity RD was consistently larger than the number of voxels with increased FA. The voxels indicating FA and diffusivity changes were bilaterally distributed, but with a left-sided preponderance in the striatum (Fig. 4b, b′).

The voxel-based analysis thus confirmed presence of significantly increased FA in the ventral striatum, GP, EP, and SN, and decreased diffusivity in the GP and EP in HD rats. The voxel-based analysis further indicated presence of decreased MD and RD, as well as some clusters with increased RD, in the ventral striatum and frontal cerebral cortex. In addition to the regions included in the hypothesis-driven ROI comparison, the voxel-based analysis indicated FA and diffusivity changes in the hippocampus, thalamus, and subthalamic region, all of which are associated with HD (Braak and Braak 1992; Vonsattel et al. 1985; Vonsattel and DiFiglia 1998; Rosas et al. 2006; Della Nave et al. 2010; Hobbs et al. 2010).

## Discussion

We have compared high-resolution ex vivo structural MRI and DTI measurements from aged transgenic HD and wild-type rats, and provide by two independent analyses evidence of altered DTI measurements in specific HD relevant brain regions. Our findings extend earlier efforts at characterizing the morphological phenotype of HD rats, and confirm that DTI is a promising method for monitoring HD-related tissue changes in this model.

### Validity of findings

Our DTI data were obtained ex vivo from fixed tissue with the use of a paramagnetic contrast medium. While anisotropy measures are relatively stable in fixed specimens, diffusivity measures are considerably reduced (Guilfoyle et al. 2003; Sun et al. 2003, 2005; Zhang et al. 2012). The same is found with the use of contrast enhancement (D’Arceuil et al. 2007). It should be noted that reduced diffusion occurring in fixed tissues may influence signal attenuation, which at the employed diffusion weighting (*b* = 800 s/mm^2^) in theory can bias diffusion sensitivity towards the fast diffusion component in the tissue. Since multiple factors, such as tissue fixation, temperature, and the employed acquisition scheme will have complex influences on DTI measures (Shepherd et al. 2009), considerable care should be taken when extrapolating our ex vivo findings to in vivo values. The observed group differences should nevertheless be valid, since any methodological bias would have similar effect on the two groups compared.

Morphological distortions related to the ex vivo procedure were minimized by gentle perfusion and scanning with brains in situ within the cranium. The high spatial resolution and structural contrast obtained with our ex vivo MR images greatly facilitated the anatomical segmentation of the regions investigated, which was particularly important for delineating the GP, EP, and SN with little partial voluming. In this respect, the here employed ex vivo approach has a clear advantage over in vivo investigations, albeit at cost of precluding longitudinal studies and introducing bias by tissue fixation (Lerch et al. 2012; Zhang et al. 2012). An advanced co-registration procedure was used for the voxel-based statistics, and spatial registration was found to be very good, in particular in the forebrain and basal ganglia regions. In morphologically complex regions, such as the hippocampus and brain stem, co-registration may in theory be less optimal, which potentially could influence findings in these regions. Since we used inbred animals, background heterogenetity is unlikely to have contributed to variability.

The average ROI measurements are prone to be diluted by partial inclusion of normal tissue, which gives a probability for false-negative findings, in particular in large regions such as the dorsal striatum. Partial volume effects by inclusion of surrounding tissues are less likely due to the small voxel size used. The relatively low number of samples used in our study was suboptimal and implies a risk for false-negative results in the voxel-based statistical comparison, and (Davatzikos 2004), as well as reduced likelihood of reaching statistical significance in the ROI-based comparisons of means. Despite this, both analyses showed changes in HD relevant regions (in line with a priori hypotheses) with most prominent DTI changes located in the ventral striatum and globus pallidus.

### Manifestations of neuropathology in HD rats

Atrophy of the caudate and putamen, cerebral cortex, thalamus, and associated white matter are pathognomonic MRI findings in clinically manifest HD (Bohanna et al. 2008), and therefore also expected in aged HD rats. But while striatal atrophy has been reported in earlier generations of transgenic HD rats (von Hörsten et al. 2003; Kantor et al. 2006; Nguyen et al. 2006), later studies have reported little or no evidence of atrophy (Winkler et al. 2006; Blockx et al. 2011). Our morphometric results confirm that there is little evidence of atrophy in HD rats, even at 18 months of age. Interestingly, we also observed an insignificantly smaller average lateral ventricle volume in HD rats, which is opposite to findings in HD patients (Bohanna et al. 2008), but in agreement with the observations by Blockx et al. (2011), who interpreted this as a possible “pathological enlargement” phenomenon (Paulsen et al. 2006). Given the absence of ‘hallmark’ striatal atrophy in transgenic HD rats, further investigations are warranted to clarify the relevance of the model for striatal degeneration in HD patients.

Regardless of the subtle morphometric findings, our DTI analyses revealed significantly elevated anisotropy and reduced diffusivity in the striatum, GP, EP, SN, thalamus, subthalamic region, and hippocampus of HD rats. These brain regions have all been associated with neuropathology in HD patients (Braak and Braak 1992; Harper 1996; Vonsattel and DiFiglia 1998; Rosas et al. 2006, 2010; Bohanna et al. 2008; Della Nave et al. 2010; Hobbs et al. 2010). Our finding of both significant volume reduction and altered DTI measurements in the ventral striatum and GP of HD rats is particularly interesting in light of earlier reported accumulation of *huntingtin* aggregates in the same regions (Nguyen et al. 2006; Petrasch-Parwez et al. 2007), and lends support to the notion that limbic systems may be affected in HD rats. Polyglutamine aggregates have previously been shown to accumulate in multiple brain regions in aged HD rats (Nguyen et al. 2006), including the regions in which we found altered DTI measurements. Our DTI findings thus correlate with earlier evidence implicating the different regions of the basal ganglia, as well as the hippocampus in the neuropathological process in HD rats. While histological studies of this model also have revealed pathology in different parts of the cerebral cortex (Nguyen et al. 2006; Kantor et al. 2006; Petrasch-Parwez et al. 2007), only limited frontal cortical AD changes were detected by our voxel-based analysis. This might be a matter of methodological sensitivity.

Our observations fit remarkably well with a priori expectations about the spatial distribution of findings, and have considerable similarities with many, but not all, DTI findings reported in HD patients. Increased FA has been demonstrated in the striatum (caudate–putamen complex; Rosas et al. 2006; Kloppel et al. 2008; Douaud et al. 2009) as well as in the internal capsule (Rosas et al. 2006) at preclinical and clinical stages of HD. Altered diffusivity (with both decreased and increased values) has been demonstrated in the putamen of HD patients (Mascalchi et al. 2004), and in the caudate nucleus of preclinical HD patients (Mandelli et al. 2010). Although extensive DTI changes have been reported in white matter of HD patients (Reading et al. 2005; Rosas et al. 2006; Kloppel et al. 2008; Della Nave et al. 2010; Mascalchi et al. 2004), similar findings have not been revealed in adult transgenic HD rats (Blockx et al. 2011; present study), and only to some extent in young HD rats (Blockx et al. 2012). This lack of measurable white matter DTI alterations in HD rats might relate to methodological sensitivity, but more likely indicates that white matter changes are not a profound effect in the HD rats. Indeed, evaluation of corticostriatal and striatofugal connections in aged transgenic HD rats by axonal tracing and immunohistochemistry show largely intact circuits (Van Dongen et al. 2010). Whether this indicates that this ‘late onset’ transgenic HD rat model rather mimics early stage HD or displays a different pathological process remains to be elucidated.

### On the interpretation of DTI measurements

Interpretation of DTI measurements is not trivial, as these relate to microscopic level water diffusion which is influenced by several tissue parameters, including the orientation, coherence, and integrity of neural fibers, as well as fiber myelination, and cell packing densities (Beaulieu 2002; Hagmann et al. 2006; Alexander et al. 2007). In consequence, neuropathological processes, such as neuronal or axonal loss, gliosis or inflammation may have competing influence on DTI measurements. An important limitation of the DTI technique is that it assumes a Gaussian distribution of water diffusion. With this model, DTI measurements perform well in regions containing homogeneously oriented (white matter) tissue architectures (Kaufman et al. 2005), but not in regions with crossing fiber orientations (Beaulieu 2002; Leergaard et al. 2010). In regions with coherent white matter, DTI measures thus have predictable relations to neuropathologies such as ischemic stroke, demyelination, inflammation, and edema (reviewed in Alexander et al. 2007). In grey matter and as well as in regions with crossing fiber orientation, however, changes in DTI parameters following neuropathology will likely depend on the underlying tissue architecture. Given these limitations of DTI, alternative diffusion MRI models measuring non-Gaussian water diffusion, such as diffusion kurtosis (Jensen et al. 2005; Veraart et al. 2011b), may be more sensitive for probing microstructural changes in regions with complex tissue architectures (Wu and Cheung 2010; Blockx et al. 2012).

Earlier observations of DTI alterations in HD patients fit well with our findings. Increased FA has been described in the putamen and GP of early stage HD patients (and presymptomatic carriers), and tentatively interpreted as modified tissue integrity, such as neuronal remodeling, astrocytosis, and loss of specific axonal connections (Rosas et al. 2006). Similarly, increased FA (and MD) in symptomatic HD patients has been correlated with measurements of reduced fiber dispersions, indicating loss of specific radiating striatopallidal connections (Douaud et al. 2009). Reduced diffusivity seen after experimentally induced degeneration in the nigrostriatal tract has been interpreted to reflect reactive gliosis (Van Camp et al. 2009). There is, however, some variance with respect to the nature of DTI changes reported in rat models of HD. In vivo DTI imaging of the quinolinic acid rat model of Huntington’s disease showed reduced FA and increased diffusivity (MD, AD, and RD) in response to pronounced neurodegeneration (Van Camp et al. 2012). In addition, longitudinal in vivo DTI measurements in transgenic HD rats showed different age-related changes of DTI parameters in white matter of HD rats relative to WT controls and a trend towards increased in MD in the striatum and GP (Blockx et al. 2011), while our comparison (at one time point in older animals) revealed no distinct DTI changes in white matter and a trend towards decreased diffusivity in the striatum. In a recent in vivo diffusion kurtosis imaging study of early developmental changes in young transgenic HD rats, increased diffusivity measures were reported in the cerebral cortex and external capsule, while reduced axial kurtosis correlated to reduced myelin basic protein staining was observed in the dorsal striatum (Blockx et al. 2012). Although these findings contrast the apparent lack of pronounced DTI changes in the cerebral cortex and dorsal striatum of much older rats (present study), the results are difficult to compare since maturation and aging effects, as well as different methodological sensitivity and choice of region of interest will influence the results. While some neuropathological parameters have been charted in transgenic HD rats (Nguyen et al. 2006; Petrasch-Parwez et al. 2007), and some correlations between histological and MRI measures are established (Blockx et al. 2012), our understanding of neuropathological changes occurring across the lifespan of transgenic HD rats remains insufficient, and more specific and quantitative characterization of such changes at different ages is needed to support interpretations of neuroimaging data.

Variations among clinical DTI studies of HD patients and preclinical studies of rodent models may thus be ascribed to a multitude of biological and methodological factors. However, regardless of the nature of the observed DTI changes, there seems to be good consistency with respect to their spatial localization.

## Conclusion

We report evidence for DTI changes in the basal ganglia and hippocampus of aged transgenic HD rats. Owing to the high isotropic voxel resolution employed we were able to identify DTI changes in specific, small basal ganglia regions that are associated with HD. In line with several earlier investigations (Winkler et al. 2006; Blockx et al. 2011), we demonstrate that the transgenic HD rat model does not exhibit the hallmark basal ganglia atrophy seen in HD patients. Nevertheless, our DTI findings correlate well with earlier histological and neuroimaging findings in this HD model, as well as with several clinical studies of HD patients. Our findings add to the growing body of evidence that diffusion MRI measurements provide promising correlates for monitoring neuropathology in transgenic HD rats. However, further studies are needed to characterize and quantify the complex relationships between different pathological phenomena and diffusion MRI measurements.
